# Metastatic prostate cancer masquerading clinically and radiologically as a primary caecal carcinoma

**DOI:** 10.1186/1477-7819-5-2

**Published:** 2007-01-07

**Authors:** Muhammad A Kabeer, Edward Lloyd-Davies, Giles Maskell, Rolf Hohle, Joseph Mathew

**Affiliations:** 1 Department of Histopathology, Royal Cornwall Hospital, Truro, TR1 3LJ, UK; 2 Department of Surgery Royal Cornwall Hospital, Truro, TR1 3LJ, UK; 3 Department of Clinical Imaging, Royal Cornwall Hospital, Truro, TR1 3LJ, UK

## Abstract

**Background:**

Prostatic carcinoma is the second most common cause of cancer-related deaths in males in the West. Approximately 20% of patients present with metastatic disease. We describe the case of a patient with metastatic prostate cancer to the bowel presenting clinically and radiologically as a primary caecal cancer.

**Case presentation:**

A 72 year-old man presented with abdominal discomfort and a clinically palpable caecal mass and a firm nodule on his thigh, the latter behaving clinically and radiologically as a lipoma. Computed tomographic (CT) scan showed a luminally protuberant caecal mass with regional nodal involvement. The patient was being treated (Zoladex^®^) for prostatic cancer diagnosed 6 years previously and was known to have bony metastases. On admission his PSA was 245.4 nmol/ml. The patient underwent a right hemicolectomy. Histology showed a poorly differentiated adenocarcinoma which was PSA positive, confirming metastatic prostatic adenocarcinoma to the caecum. The patient underwent adjuvant chemotherapy and is free from recurrence a year later.

**Conclusion:**

Metastasis of prostatic carcinoma to the bowel is a very rare occurrence and presents a challenging diagnosis. The diagnosis is supported by immunohistochemistry for PSA. The treatment for metastatic prostate cancer is mainly palliative.

## Background

Prostatic carcinoma is the second most common cause of cancer related deaths in males in the West [1]. Approximately 20% of patients present with metastatic disease but colo-rectal involvement is rare[2]. We describe the case of a patient with metastatic prostate cancer to the bowel presenting as primary caecal cancer.

## Case presentation

A 72-year-old man presented with abdominal discomfort with small amount of bleeding per rectum and a clinically palpable lump in the right iliac fossa. He also had a large, firm, mobile lump on his left thigh.

He had been treated six years previously for prostatic carcinoma which was Gleason's grade 3+3= 6. Bony metastases had been diagnosed recently. He was on Zoladex^® ^injections.

**Figure 1:**
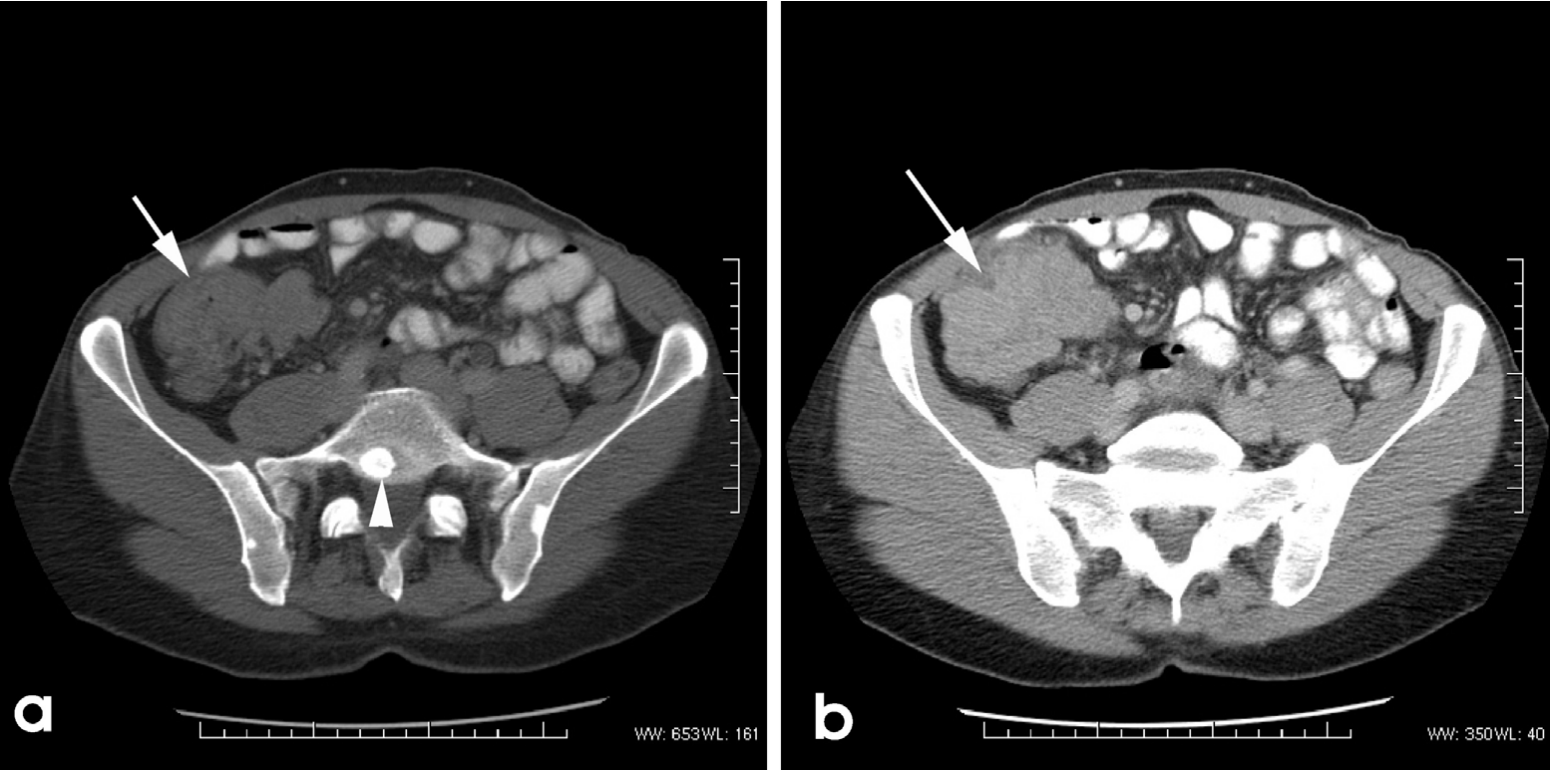
a) Imaging shows a well defined caecal tumour (arrow) with evidence of vertebral metastasis (arrowhead). b) The tumour is irregular in outline (arrow) and is closely approximated to the anterior abdominal wall.

Clinical examination showed a 6 cm hard, mobile, non-tender mass in his right iliac fossa. Digital rectal examination was unremarkable. Rigid sigmoidoscopy revealed radiation proctitis, which would explain his bleeding per rectum. CT scan of abdomen showed a caecal tumour with regional lymphadenopathy and vertebral metastasis (Figure 1). A non-palpable left iliac mass was identified: this was defined as internal iliac nodes lymphadenopathy, associated with partial obstruction of the left ureter (the possibility of prostatic metastasis to these nodes was considered). A fatty mass lesion was identified in the left thigh, the CT appearances of which were that of a simple lipoma; this has remained unchanged on subsequent CT scans. Bone scan confirmed bony metastasis in the 12^th ^thoracic and 1^st ^lumbar vertebrae and rib cage. In 2003, there was a gradual rise in his PSA levels which had gone upto 233 nmol/ml. Zoladex^® ^injections were then started and his PSA levels fell to 6.3 nmol/ml by January 2004. This however had gradually risen again to 254.4 nmol/ml on admission, inspite of being on Zoladex^®^.

**Figure 2:**
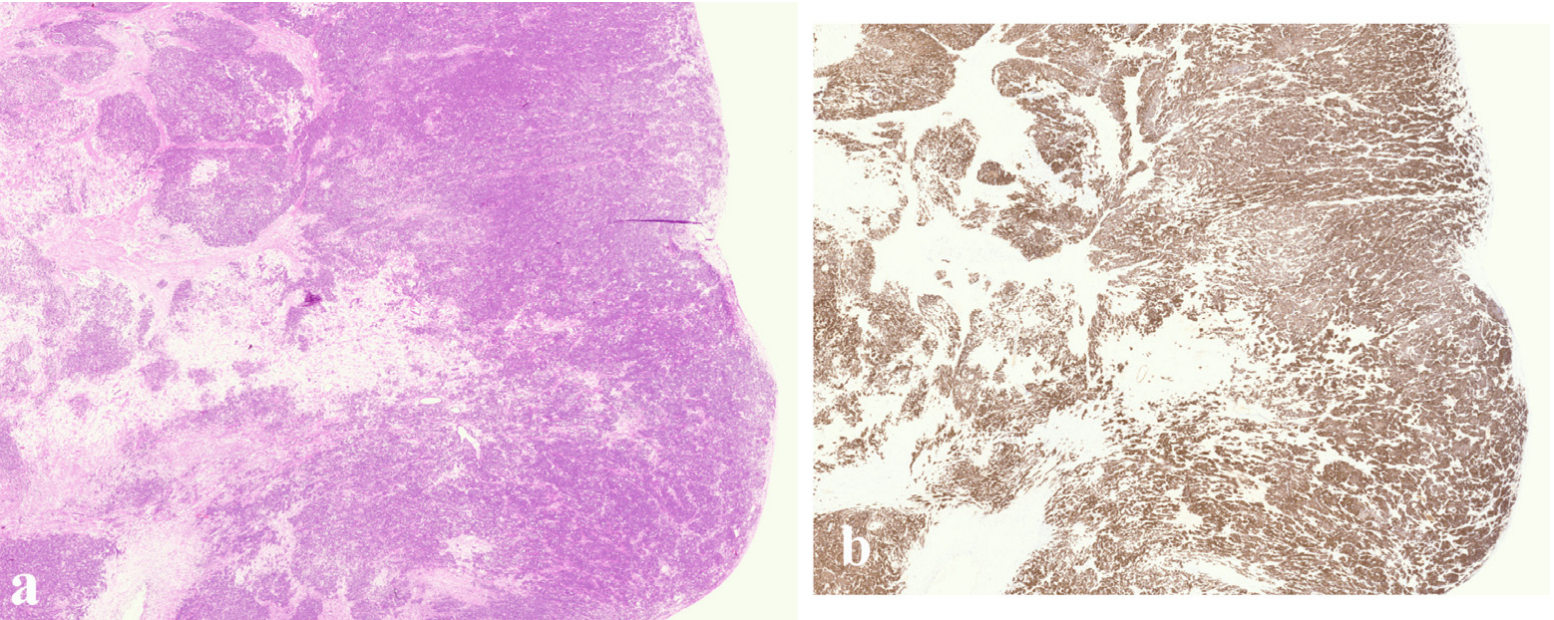
a) Histology shows a well defined intraluminal, protuberant tumour invading the submucosa. This invasive tumour extends to pericolonic fibroadipose tissue. b) This tumour is positive with PSA, confirming its metastatic origin from a primary prostatic carcinoma.

The patient underwent a right hemicolectomy for what appeared to be an obvious locally advanced luminally protuberant caecal carcinoma with mesenteric lymph nodal involvement. Histopathological examination of the specimen showed a poorly differentiated adenocarcinoma of the caecum extending throughout the whole bowel wall and breaching the serosa with metastatic involvement of 5 of 15 paracolic lymph nodes (Figure 2). The possibility of prostatic metastasis was considered and confirmed on immunohistochemical staining for PSA (Figure 2) and, given this, stains for CK7, CK20 and CDX2 immunoprofile were considered unnecessary. The final histological diagnosis was of a metastatic prostatic adenocarcinoma to the caecum. The patient underwent adjuvant chemotherapy with Docetaxel and Paclitaxel and is free from recurrence a year later.

## Discussion

Progression of prostate cancer occurs either by direct extension or metastasis through haematogenous or lymphatic routes. The most commonly involved organs are the seminal vesicles, base of the bladder, bones (mainly axial skeleton) and lymph nodes[1]. Widespread visceral involvement is quite rare. Some unusual sites of spread have been described in literature as the parotid gland[3], oesophagus[4], vocal cords[5], larynx[6], lung &bronchus[7], stomach[8], liver[9], skin[10], umbilicus [11], sphenoid sinus[12], cranium[13], meninges[14], testes[15], penis[16], breast[17], mediastinum[18], thymus [19], orbit[20], uveal tract[21], brain[22], cerebellum[23] and bones[24]. Rectal seeding on needle biopsy[25] and direct involvement occasionally occurs [26], but distant metastasis to the bowel is quite rare (prevalence 1–4% in autopsy series)[27]. So far only three accounts of metastasis to the distant bowel have been recorded, one involving the small bowel[27] and the other two involving the rectosigmoid[28,29].

It is important to distinguish primary from metastatic colorectal lesions, especially in the presence of a previous history of cancer at another site, in order to facilitate appropriate management. This is best achieved by defining the tumour type on histopathologic grounds and, in this instance, by immunohistochemical staining for PSA[17,30]. The treatment of primary colonic adenocarcinomas is potentially curative with a combination of surgery and chemo-irradiation; treatment of colonic metastasis depends on the primary site and might not be 'curative'. The treatment of metastatic disease from the prostate is purely palliative; hormonal treatment represents the standard[31], although this can be combined with debulking surgery to reduce the tumour load where feasible. However, the impact of debulking surgery on patient survival is not known. Metastatic prostate cancer has poor prognosis and survival rates range from 1 to 3 years[8].

The response of prostatic carcinoma to oestrogen therapy has been well established[32] but patients often become refractory after prolonged treatment. Options for hormone-refractory prostate cancer include secondary hormonal treatment (anti-androgens), radiotherapy and cytotoxic chemotherapy. The metastatic component, as described in our case, can be managed with debulking surgery and may well need adjuvant chemotherapy or secondary hormonal treatment to achieve reasonable regression of disease.

## Conclusion

Metastasis of prostatic carcinoma to the bowel is a very rare occurrence and presents a challenging diagnosis. The mainstay of diagnosis is histopathology supported by immunohistochemistry for PSA.

Palliative treatment remains the mainstay of therapy for metastatic prostate cancer and hormonal therapy represents the standard with debulking surgery where feasible.

## Competing interests

The author(s) declare that they have no competing interests.

## Authors' contributions

**MAK **– research, acquisition of data, acquisition of consent, writing, drafting and revision of manuscript

**ELD **– lead colorectal surgeon involved in this patients management and critical review of manuscript

**GM **– consultant in charge of imaging and diagnosis, contribution of images, critical review of manuscript

**RH **– reporting pathologist, involved in discussions leading to manuscript preparation, contribution of data and critical review of manuscript

**JM **– lead colorectal pathologist, involved in diagnosis, contributed to manuscript conception, involved in collating material, organising manuscript, critical reviews of manuscript

All authors have read and approved the final manuscript
